# Therapy-Induced Senescence: Novel Approaches for Markers Identification

**DOI:** 10.3390/ijms25158448

**Published:** 2024-08-02

**Authors:** Francesco Pacifico, Fulvio Magni, Antonio Leonardi, Elvira Crescenzi

**Affiliations:** 1 Istituto per l’Endocrinologia e l’Oncologia Sperimentale, CNR, Via S. Pansini 5, 80131 Naples, Italy; f.pacifico@ieos.cnr.it; 2 Proteomics and Metabolomics Unit, Department of Medicine and Surgery, University of Milano-Bicocca, 20854 Vedano al Lambro, Italy; fulvio.magni@unimib.it; 3 Dipartimento di Medicina Molecolare e Biotecnologie Mediche, University of Naples “Federico II”, Via S. Pansini 5, 80131 Naples, Italy; leonardi@unina.it

**Keywords:** therapy-induced senescence, SASP, biomarkers, senotherapy, senolytics

## Abstract

Therapy-induced senescence (TIS) represents a major cellular response to anticancer treatments. Both malignant and non-malignant cells in the tumor microenvironment undergo TIS and may be harmful for cancer patients since TIS cells develop a senescence-associated secretory phenotype (SASP) that can sustain tumor growth. The SASP also modulates anti-tumor immunity, although the immune populations involved and the final results appear to be context-dependent. In addition, senescent cancer cells are able to evade senescence growth arrest and to resume proliferation, likely contributing to relapse. So, research data suggest that TIS induction negatively affects therapy outcomes in cancer patients. In line with this, new interventions aimed at the removal of senescent cells or the reprogramming of their SASP, called senotherapy, have become attractive therapeutic options. To date, the lack of reliable, cost-effective, and easy-to-use TIS biomarkers hinders the application of recent anti-senescence therapeutic approaches in the clinic. Hence, the identification of biomarkers for the detection of TIS tumor cells and TIS non-neoplastic cells is a high priority in cancer research. In this review article, we describe the current knowledge about TIS, outline critical gaps in our knowledge, and address recent advances and novel approaches for the discovery of TIS biomarkers.

## 1. Therapy-Induced Senescence

Therapy-induced senescence (TIS) is a state of cellular senescence induced by anticancer treatments in both cancer cells and normal, non-neoplastic cells residing in the tumor microenvironment. The first observation of TIS dates back to 1999, when Chang and colleagues described the ability of different chemotherapeutic drugs and ionizing radiation to induce in human tumor cell lines morphological and biochemical changes resembling the replicative senescence observed in normal cells [1]. While p53 is not essential for TIS, which can be also induced in p53-null and p53-mutant cancer cells [2,3], single-cell analysis has revealed that high levels of p21 expression at early time points lead to a final fate of cellular senescence after chemotherapy [4]. Interestingly, early studies showed that different chemotherapeutic agents, with distinct mechanisms of action, have different abilities to induce TIS, as evidenced by the relative increase in the percentage of cells positive for a widely used senescence marker, the senescence-associated beta-galactosidase (SA-beta-gal) [1]. These results were further confirmed using a larger panel of senescence markers [5]. Most importantly, the induction of tumor cell senescence after chemotherapy or radiotherapy was observed in human cancers in vivo, and TIS is now considered a relevant outcome of anticancer treatments [6,7,8,9].

As for normal cells undergoing replicative senescence, TIS cancer cells do not proliferate but they are metabolically active and undergo senescence-specific metabolic reprogramming. Their metabolism is geared to growth, resulting in enlarged cell morphology, which represents one of the hallmarks of senescence [10], and to the production of a specific secretome referred to as SASP (senescence-associated secretory phenotype) [6]. Cellular enlargement has been causally linked to the induction of CDK inhibitors p21 and p16, which arrest the cell cycle but do not restrain growth-promoting pathways, thereby inducing cellular hypertrophy and geroconversion, i.e., the conversion from a transient cell cycle arrest to a permanent, senescence-like arrest [11]. In line with this, recent findings suggest that large cells size may be a cause rather than just a consequence of senescence. Increased cell size, in fact, remodels the proteome of proliferating cells towards a senescence-like state [12]. Other cellular organelles undergo senescence-specific alterations. Morphological alterations in the nucleus have been recognized for a long time as specific features of cell senescence and have recently been used as predictors of senescence [13]. The enlargement of the lysosomal compartment correlates with increased expression of the senescence-associated beta-galactosidase, which represents a senescence-specific trait [14]. Mitochondrial volume also appears to increase proportionally with cellular volume in senescent cells, a change accompanied by extensive reprogramming of mitochondrial proteome [15] and by mitochondrial dysfunctions [16]. However, mitochondrial dysfunctions are compensated by the increased mitochondrial mass [17] such that the bioenergetic activity per mitochondria is decreased but the whole mitochondrial bioenergetic function in senescent cells is enhanced [15].

As previously mentioned, TIS cancer cells acquire a SASP and secrete cytokines, chemokines, growth factors, and extracellular proteases that affect cellular and non-cellular components in the tumor microenvironment. SASP composition is heterogeneous and varies between cell types, whereas it is less influenced by the senescence inducer [18]. p53 can critically modulate both the composition and the strength of the SASP [6].

It is important to note that although TIS shares many features with replicative senescence, the timescale of senescence induction is radically different since replicative senescence in human cells requires several population doublings in vitro, while a short exposure to anticancer drugs or radiation rapidly induces TIS. The correct timing for the development of a fully senescent phenotype in vitro, and thus the appropriate temporal scale for experimental analyses, appears to be controversial [19]. Cellular senescence develops gradually, over several days. Hence, geroconversion requires time after induction [6,20], and a drug-induced cell cycle arrest cannot be considered senescence yet [11]. These observations suggest that experimental efforts should be made in order to find the appropriate cell culture conditions to unambiguously induce TIS and to avoid incomplete, abortive senescence in in vitro studies. Accordingly, a reliable assessment of cellular senescence in vitro requires verification of both primary senescence markers, which substantiate the halt of the cell cycle and distinctive morphological changes, as well as secondary markers such as the SASP [21].

In addition to classical chemotherapeutic agents and radiation, molecular targeted therapeutics represent a recent, successful tool in cancer treatment [22,23]. Molecular targeted therapeutics mainly comprise small molecule agents or monoclonal antibodies directed towards proteins overexpressed, deregulated, or mutated specifically in cancer cells and essential for cancer cell survival. Molecular targeted therapeutics thus enable personalized treatment of tumors by targeting tumor-specific alterations, reducing side effects in patients. A variety of molecular targeted drugs have been shown to simultaneously induce apoptosis and TIS in cancer cells in vitro and in vivo [24,25]. Interestingly, molecular targeted therapeutics specifically developed to induce senescence in cancer have recently been identified, and the efficacy of this pro-senescence approach has been validated. For instance, WM-8014 and WM-1119, two selective inhibitors of histone acetyltransferases KAT6A/B, which suppress cellular senescence, were shown to reactivate a senescence program controlled by p16INK4A and p19ARF. WM-1119, tested in vivo because of better bioavailability, effectively prevented the progression of lymphoma in mice [26]. By using a kinome-focused genetic screen, Wang and colleagues identified XL413, a potent inhibitor of the DNA-replication kinase CDC7, which induced senescence selectively in liver cancer cells with mutations in TP53 [27]. Colucci and colleagues through a chemogenomic screen identified adapalene, a retinoic acid receptor agonist, which his able to induce senescence in prostate cancer cells [28].

Cancer treatments affect both malignant and non-malignant cells in the tumor microenvironment, with different possible outcomes. Classic chemotherapeutic agents specifically target rapidly proliferating cells and induce either apoptosis or senescence in cancer cells. Notably, induction of TIS occurs independently of p53 status. Quiescent cancer cells are naturally resistant to such treatments, accumulate after treatments, and can repopulate the tumor [29]. In contrast, classic chemotherapy agents fail to induce apoptosis in stromal cells. Chemotherapy mainly results in the remodeling of normal, resident fibroblasts and their conversion to activated, pro-tumorigenic CAF [30,31]. SASP factors secreted from senescent tumor cells also contribute to reprograming the phenotype of local fibroblasts to CAF. Finally, CAF response to chemotherapy is highly variable, but innate resistance to apoptosis induced by different cytotoxic agents has been described [32]. In contrast, CAF readily undergo p53-mediated senescence in response to chemotherapy [33]. Hence, cancer cells, normal fibroblasts, and CAF show different cell fates in response to chemotherapy. Similar differences have been reported in response to radiation therapy: CAF are resistant to radiation-induced apoptosis, but susceptible to radiation-induced senescence. Radiation can drive the conversion of normal fibroblasts into activated CAF [34]. Finally, radiation therapy induces various responses in cancer cells, ranging from apoptosis to senescence and mitotic catastrophe [35].

**Figure 1:**
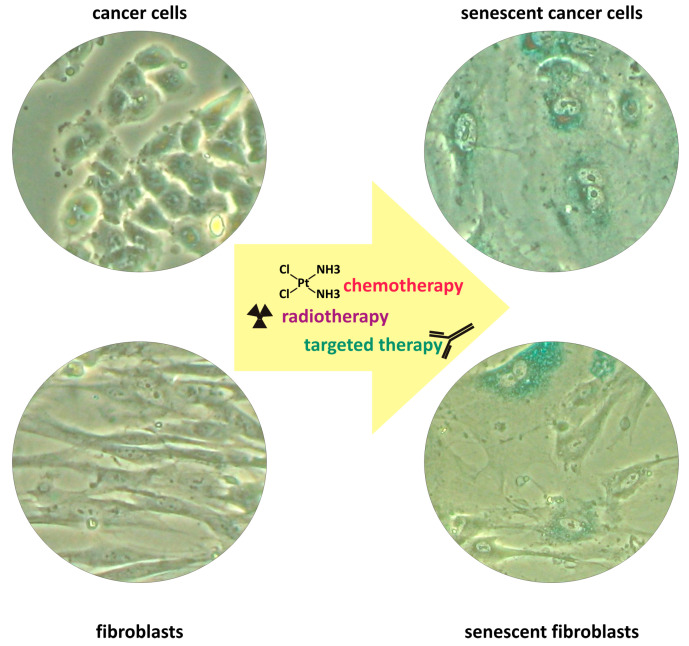
Representative morphological alterations and SA-beta-gal staining in therapy-induced senescent malignant and non-malignant cells.

In conclusion, cellular senescence is a common response activated in both cancer and non-malignant cells in response to classical therapeutic approaches, as well as in response to targeted therapeutic strategies (Figure 1).

Furthermore, the feasibility of identifying therapeutic agents able to selectively induce cellular senescence has been demonstrated. However, how TIS contributes to the outcome of cancer therapy is still a matter of research, since on the one hand, TIS activates anti-tumor immunity, but on the other hand, TIS cells sustain several hallmarks of cancer, as discussed in the next sections.

## 2. Favorable Effects of TIS on Anti-Tumor Immunity

The first indications that senescent cells can activate the innate immune system to limit tumor growth came from a mouse model of p53 reactivation in p53-deficient liver carcinoma. The primary response to reactivation of the p53 pathway was not apoptosis but cellular senescence, and this response not only produced a strong cell cycle arrest but also induced the upregulation of inflammatory cytokines, the activation of an innate immune response, and tumor clearance [36]. Similarly, in a mouse model of liver carcinoma, inducible p53 expression activated a senescence program, with the development of a SASP rich in chemokines, which recruited natural killer (NK) cells and drove tumor clearance [37].

A positive effect of TIS on anti-tumor immunity has also been reported in the context of chemotherapy, where the induction of senescence promoted antigen presentation and expanded the repertoire of antigens presented. The authors also demonstrated that senescent cancer cells showed a higher immunogenic potential as compared to non-cancer TIS cells [38]. The immunogenic phenotype of TIS cells is related to a senescence-specific alteration of the surface proteome, with upregulation of the positive regulators of immune signaling, such as IFN-gamma signaling effectors, which increase TIS cells’ visibility to the adaptive immune system [39]. Similarly, in a mouse model of multiple myeloma, TIS enhanced surface expression of the NKG2D ligands RAE-1 and MULT-1, leading to tumor cell recognition and killing by NK cells [40]. The SASP has also been shown to induce Fas/CD95 upregulation in TIS cancer cells in vitro, thereby selectively enhancing susceptibility to Fas-mediated apoptosis [41]. Interestingly, autophagy inhibition has recently been shown to potentiate the effects of chemotherapy-induced senescence on the anti-tumor immune response in glioblastoma in vivo [42]. Many targeted therapeutics that induce TIS have also been shown to activate anti-tumoral immunity. For instance, the Aurora kinase inhibitor MLN8237 mainly induced TIS in human melanoma implants, and SASP factors from TIS neoplastic cells recruited both macrophage and neutrophil [43]. A combination of MEK and CDK4/6 inhibitors, targeting oncogenic KRAS, induced senescence in pancreatic ductal adenocarcinoma cells and preclinical mouse models. The SASP developed by these TIS cells triggered vascular remodeling and increased infiltration of CD8+ T cells [44]. XL413, a recently identified inhibitor of CDC7 kinase, induced senescence in p53-mutant liver cancer cells and promoted tumor infiltration with macrophages and T cells [27]. Combination therapy with adapalene and docetaxel not only induced senescence in prostate cancer cells but also activated NK-mediated tumor clearance [28]. The CDK4/6 inhibitor abemaciclib induced TIS in mouse models of breast and colorectal carcinoma and boosted tumor cells immunogenicity [45]. Notably, in this study, abemaciclib-induced cellular senescence developed without a canonical SASP, suggesting that higher immunogenicity was not SASP-dependent [45].

**Figure 2:**
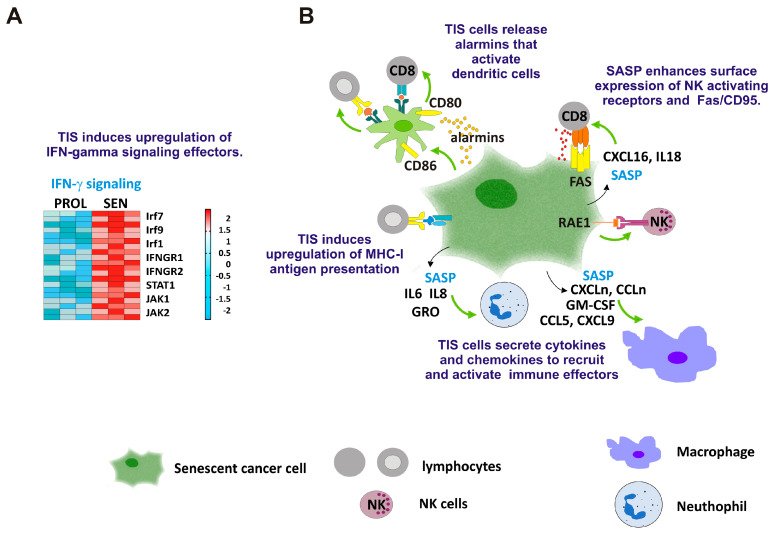
TIS cells can stimulate anticancer immunity. (**A**) Representative heat map illustrating changes in the expression of type II IFN signaling components in proliferating (PROL) versus senescent (SEN) cells, analyzed in triplicate. The color bar represents different enrichment scores. Blue: negative; red: positive. (**B**) TIS cells release alarmins and are highly efficient in activating dendritic cells. TIS cells secrete both cytokines and chemokines (CXCLn, CCLn) that can recruit and activate T lymphocytes, macrophages, and neutrophils. TIS also enhances the surface expression of NK-activating receptors, as well as Fas/CD95. Black arrows indicate the release of SASP components from senescent cells; green arrows indicate positive stimuli to immune cells.

On the whole, these studies indicate that TIS can stimulate anticancer immunity, although the immune populations activated, the signaling pathways involved, and the final outcome appear to be context-dependent (Figure 2).

The ability of TIS cells to activate anti-tumor immune responses can also sensitize tumor cells to immunotherapy. For instance, cellular senescence induced by CDK4/6-targeted drugs in melanoma was shown to repress a resistance program expressed by malignant cells prior to immunotherapy, which is associated with T cell exclusion and predicts unfavorable clinical response to anti-PD-1 therapy [46]. Interestingly, Hao and colleagues devised an experimental strategy by which to boost the SASP ex vivo, which sensitized tumor cells to immune checkpoint blockade [47].

However, immune clearance may be hampered by excessive accumulation of senescent cells, which outnumber recruited immune cells. In addition, the SASP changes its composition over time, possibly fostering polarization of immune cells towards a tumor-promoting phenotype and/or inducing immune dysfunction. Below, we discuss the adverse effects of TIS.

## 3. Adverse Effects of TIS

**Figure 3:**
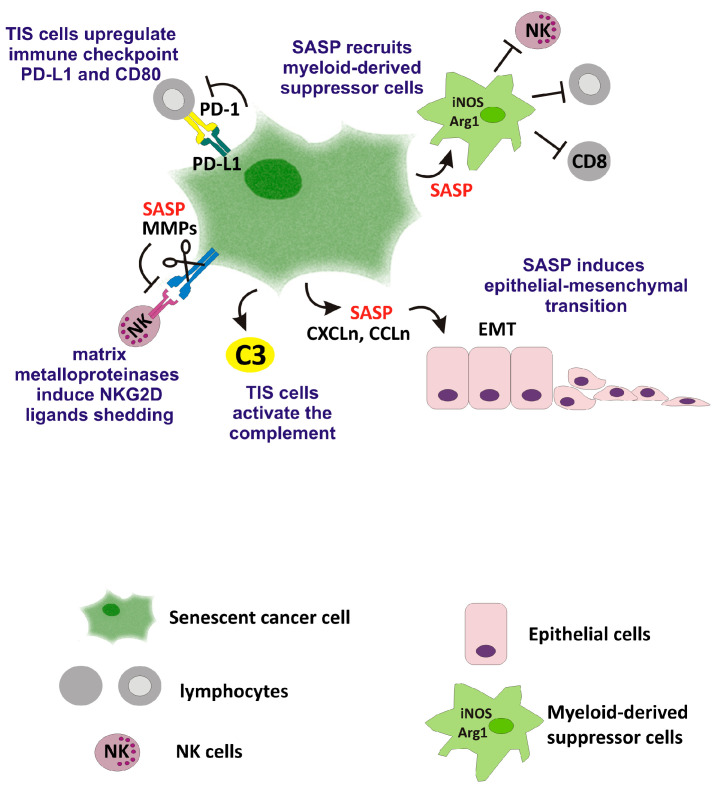
Adverse effects of TIS cells. SASP components induce epithelial–mesenchymal transition (EMT) in epithelial cells and accumulation of myeloid-derived suppressor cells, which suppress T-cell activity. SASP components such as matrix metalloproteinases (MMP) induce NKG2D ligands shedding and NK cells inactivation. TIS cells also upregulate immune-inhibitory proteins (PD-L1). Finally, TIS cells activate the complement (C3).

Although available studies indicate that TIS cells can activate anti-tumor immunity, several SASP components have been shown to foster cancer cell proliferation, invasion, and plasticity and to hamper anticancer immunity (Figure 3). For instance, in a MMTV-Wnt1 breast tumor model, the presence of WT p53 was associated with the induction of TIS and the development of a SASP enriched in eotaxin, Cxcl5, and RANTES, which induced proliferation in neighboring tumor cells [48]. The SASP from TIS colorectal cancer cells induced epithelial–mesenchymal transition (EMT) and invasiveness in proliferating cancer cells [8]. Likewise, the SASP developed from non-malignant, normal senescent fibroblast has been shown to induce EMT and invasiveness in non-aggressive human breast cancer cell lines [6].

More recently, the SASP has also been implicated in the negative modulation of the anti-cancer immune response. For instance, TIS fibroblasts induced by the CDK4/6 inhibitor palbociclib in an immunocompetent murine model promoted not only melanoma cell proliferation but also the accumulation of myeloid-derived suppressor cells [49]. The SASP of senescent cancer-associated fibroblasts has been mostly associated with chronic inflammation, increased cancer aggressiveness, and immunosuppression, as recently reviewed [50]. Furthermore, senescent fibroblasts upregulated the immune checkpoint molecule PD-L1, which drove immune cell inactivation [51]. Shahbandi and colleagues, using a p53 WT mouse mammary tumor model, investigated changes in both immune-modulatory genes and checkpoints expressed by chemotherapy-induced senescent tumor cells. They demonstrated that TIS cells surviving in residual disease after neoadjuvant chemotherapy upregulated genes related to antigen processing and presentation, thus supporting the idea that TIS tumor cells are easily detected by the immune system. TIS cells, however, also upregulated the immune-inhibitory proteins PD-L1 and CD80 [52]. The SASP can also favor immunoevasion from NK cells. TIS fibroblasts upregulated the NKG2D ligands MICA and MICB, which mediate immune recognition and clearance by NK cells, but a subset of TIS cells evaded NK recognition. These resistant cells developed a SASP enriched in matrix metalloproteinases that induced NKG2D ligands shedding and caused immune evasion. Importantly, these processes were detected in residual, drug-resistant tumors from prostate and breast cancer patients treated with senescence-inducing genotoxic chemotherapies [53].

Induction of TIS in cancer cells led to the upregulation and activation of another component of the immune system, the complement. Importantly, increased C3 expression was also observed in breast cancer samples expressing TIS hallmarks following exposure to neoadjuvant chemotherapy [54]. The authors hypothesized that secreted complement proteins participate in the enhancement of harmful inflammation in the tumor microenvironment.

Overall, these data indicate a critical role for senescence and the SASP in determining the therapy outcome in cancer. Notably, interventions aimed at modifying the SASP may switch a tumor-suppressive response into a tumor-promoting response [55,56]. These data, on the whole, support the idea that the presence of TIS cells, either malignant or non-malignant, could be detrimental, and highlight the clinical potential of senescent cells removal in cancer, which was substantiated using a genetic approach. Using a p16INK4a transgenic mouse model (p16-3MR) that allows for both bioluminescent visualization and the selective elimination of senescent cells in living animals, Demaria and colleagues demonstrated the induction of TIS in vivo in response to chemotherapeutic drugs. When TIS breast cancer cells were eliminated by ganciclovir treatment, cancer relapse and metastatic spread were greatly reduced, whereas selective elimination of normal TIS cells reduced several debilitating side effects of the chemotherapy [57].

Finally, it is worth recalling an additional potential harmful effect of TIS: the ability of senescent cancer to evade senescence, re-enter the cell cycle, and resume proliferation [58]. Escape from TIS represent a challenge in cancer treatments, likely favoring tumor relapse [59]. Senescence evasion is a rare event driven by a small subset of plastic cancer cells characterized by a stem cell-like phenotype [3,60]. Interestingly, a direct link between the activation of premature senescence and the reprogramming of bulk tumor cells into cancer stem cells (CSC) has been demonstrated [61].

In conclusion, the studies described demonstrate that TIS is a common response to both classical therapeutic approaches and to targeted therapeutic strategies, as summarized in Table 1. TIS cells, on the one hand, are able to activate anti-tumor immune responses, but on the other hand, they can lead to the enhancement of inflammation in the tumor microenvironment, immunosuppression, and increased malignancy. So, the development of new therapeutic strategies to counteract the detrimental effects of TIS is a novel research frontier, as illustrated in the next section.

## 4. Therapeutic Approaches to Target TIS Cells

The studies discussed demonstrate that both malignant and non-malignant TIS cells in the tumor microenvironment may be harmful for cancer patients. The beneficial and adverse effects of cellular senescence representing more than two alternatives likely represent two consecutive steps: the induction of cellular senescence as an acute event, readily followed by immune-mediated clearance, mediates favorable effects; whereas the failure of senescent cells removal and the resulting chronic accumulation of senescent cells promotes adverse effects. In the context of cancer, the available data suggest that detrimental effects prevail. Hence, the removal of senescent tumor cells or the reprogramming of their SASP, called senotherapy, has become an attractive option to take in preventing senescence-induced tumor progression [62]. Drugs that suppress or modulate the SASP have been studied, referred to as senomorphics, and senolytic agents that selectively induce apoptosis in senescent cells have been developed, as recently reviewed [63]. Both senomorphic and senolytic agents can synergize with conventional or molecularly targeted cancer therapies. For instance, the histone deacetylase inhibitor panobinostat was evaluated as a senolytic drug in chemotherapy-induced senescent non-small-cell lung cancer and head and neck squamous cell carcinoma cells. Panobinostat decreased expression of Bcl-XL and caused cell death in TIS cancer cells [64]. In rodent models of mitoxantrone-induced TIS, the flavonoid procyanidin C1 (PCC1) inhibited SASP development at low concentrations, while selectively depleting TIS cells at higher concentrations, thereby enhancing the efficacy of chemotherapy [65]. By using mouse models of prostate cancer characterized by the presence of both proliferative and senescent components and single-cell RNA-sequencing, Troiani and colleagues identified the antiapoptotic protein Mcl-1 as a key survival factor in senescent tumor cells. In line with this, a pharmacological inhibitor of Mcl-1 acted as a senolytic and enhanced the efficacy of docetaxel [66]. The senolytic drug ABT-263 (navitoclax, a selective inhibitor of BCL-2 family proteins) selectively eliminated TIS prostate and ovarian fibroblasts and interfered with senescence-induced malignant phenotype of cancer cells [67]. Similarly, administration of navitoclax after cranial irradiation eliminated radiation-induced senescent brain astrocytes in vivo, which promoted glioma cells migration and invasion via paracrine secretion of HGF, and so attenuated glioblastoma recurrence [68]. By performing an unbiased RNA sequencing on primary human fibroblasts induced to undergo senescence via ionizing radiation, Baar and colleagues discovered that TIS fibroblasts are primed to undergo apoptosis, which is restrained by upregulated FOXO4. These authors designed a FOXO4 interacting peptide that perturbed FOXO4–p53 interaction and caused TIS cells apoptosis [69]. A screening of a natural medicinal agent library for senotherapeutic candidates led to the identification of rutin, a phytochemical product, that showed both senomorphic and senolytic capacity on TIS stromal cells. Rutin-dependent SASP dampening improved the outcome of chemotherapy in preclinical mouse models of prostate cancer cells [70].

**Figure 4:**
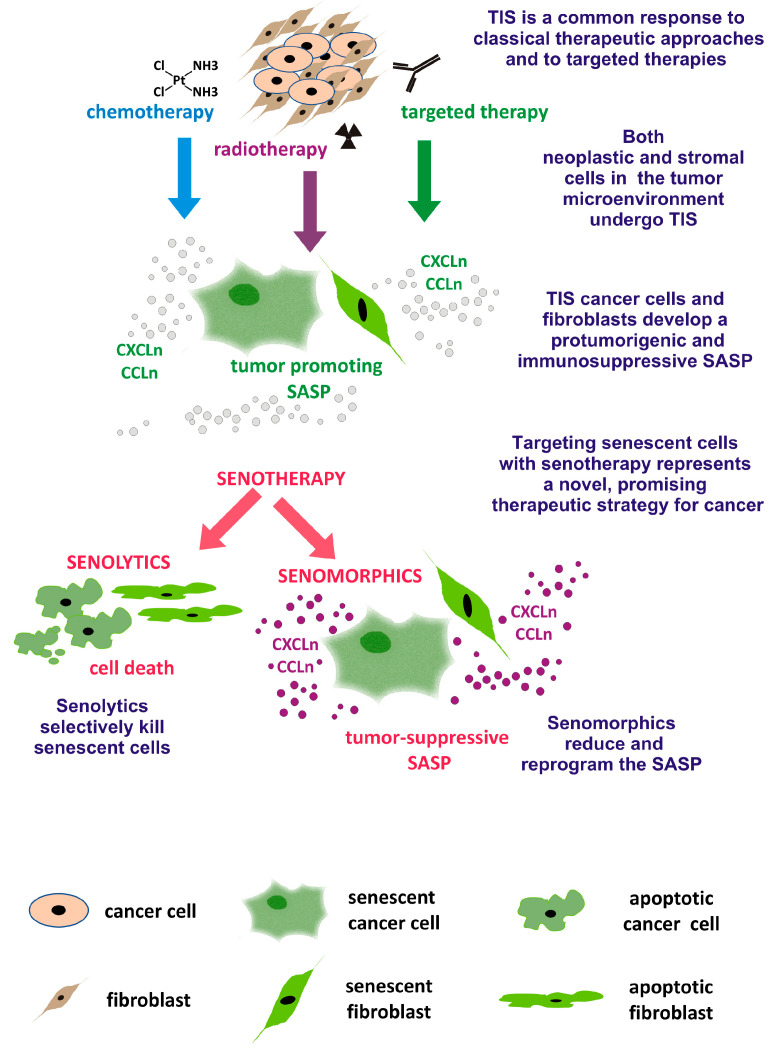
A two-hit combination therapy with TIS-inducing agents and senotherapy represents a promising therapeutic strategy for cancer. TIS is induced in both neoplastic and stromal cells in response to classical therapeutic approaches and to targeted therapies. Senolytic drugs selectively eliminate senescent cells, while senomorphics reduce and reprogram the SASP, favoring a tumor-suppressive and immune-stimulating microenvironment.

On the whole, combination therapy with either chemotherapy or radiotherapy to induce senescence, followed by senotherapy, which targets both non-neoplastic and neoplastic senescent cells in the tumor microenvironment, improves the outcome of cancer treatment and reduces side effects (Figure 4).

Thus, extensive senotherapeutics discovery efforts are ongoing, as recently reviewed [71]. Interestingly, it has recently been shown that combining high-throughput phenotypic screenings with artificial intelligence approaches (deep learning) can facilitate the discovery of new senolytic drugs [72]. However, the application of senotherapy in cancer requires suitable biomarkers for detecting the induction of cellular senescence upon therapy.

## 5. TIS Biomarkers

Much research is currently focusing on the development of biomarkers of aging for longevity interventions [73]. A similar effort is required for the identification and characterization of TIS biomarkers in cancer in order to detect the induction of cellular senescence in cancer patients reliably. The lack of specific TIS biomarkers, in fact, limits the application of senotherapy to malignant and non-malignant TIS cells in cancer patients.

It is important to note that some phenotypic and biochemical alterations are common in different types of senescent cells. For instance, increased expression and activity of the acidic SA-beta-gal has been widely used as a senescence biomarker in both non-neoplastic cells and cancer cells [1,14], although this marker is not completely specific [74]. However, analysis of senescence-specific gene expression has shown highly different profiles of expression between senescent fibroblasts and senescent epithelial cells [75,76]. Also, SASP composition has been shown to be different in different cell types [18]. These results suggest that it would be possible to identify senescence biomarkers associated with specific cell types. The identification of cell-type-specific senescence biomarkers would be of considerable use to the academic community as tools for targeting and imaging different types of TIS cells, as well as for understanding the complex biology of the tumor microenvironment.

Below, we describe the most commonly used biomarkers for senescence detection, as well as the application of novel technological approaches to TIS biomarkers discovery, such as image-based classifiers.

### 5.1. Senescence-Associated Beta-Galactosidase

The most commonly used method for the identification of senescent cells is the detection of the acidic, pH 6, active SA-beta-gal, which is not completely specific since its activity is increased in cells under a variety of stress conditions, such as serum withdrawal or confluence [74,77]. Furthermore, since the detection of the SA-beta-gal is determined as an enzymatic assay, it requires the use of fresh tissue. This limitation poses a difficulty for TIS detection in clinical specimens from cancer patients, which are routinely available as formalin-fixed and paraffin-embedded (FFPE) tissues. Conversely, the activity of SA-beta-gal has been exploited for in vivo imaging and for drug delivery through galacto-oligosaccharide conjugation or coating [78]. In these systems, SA-beta-gal expressed in senescent cells mediates the activation of imaging probes or senolytic pro-drugs through the digestion of nanoparticles and release of the encapsulated material, as recently reviewed by Morsli [79].

### 5.2. Lipofuscin

Lipofuscin has been suggested as a valuable biomarker of cellular senescence. Lipofuscin is a brown, fluorescent pigment that accumulates with age in the lysosomal compartment of cells and is essentially composed of residues of incomplete lysosomal digestion of oxidized proteins and lipids. Lipofuscin sensitivity and specificity has been validated in different senescence settings, and various detection methods for in vitro and in vivo applications have been developed. For instance, by using Sudan black B dye, Georgakopoulou demonstrates that lipofuscin accumulated in replicative senescent human cells, as well as in TIS normal and neoplastic cells. Notably, Sudan black B staining was applicable in FFPE tissue samples [80]. However, some limitations were detected. For instance, since the lipofuscin granules were very small, their visualization required high magnifications, especially in FFPE sections, rendering their identification challenging. Hence, in a second work, these authors designed a biotin-linked Sudan black B analogue, which allowed for enhanced visualization [81]. Recently, a fluorophore-conjugated Sudan black B analog, GLF16, was synthesized, which easily detected senescent cells via fluorescence microscopy and flow cytometry [82].

### 5.3. Cell Surface Markers

Cell surface proteins represent key targets for the development of biomedical applications because of their easy accessibility to probes and drugs [83]. In an attempt to find surface senescence biomarkers, Althubiti and colleagues used a classical proteomic approach and screened plasma membrane-associated proteins expressed in senescent EJ bladder cancer cells. Induction of senescence was achieved via the inducible expression of CDK inhibitors p21 or p16, and mass spectrometry was used to compare membrane-associated proteins in senescent cells versus their non-induced proliferating counterparts. The authors validated 10 proteins that could be used, singularly or in combination, to recognize senescent cells in culture and in tissue samples. Interestingly, some proteins were upregulated in either p16- or p21-induced senescent cells, whereas others were induced in both. These biomarkers were also validated in normal human fibroblasts [84]. An advanced cell surface mapping approach was recently used in EJ bladder cancer cells inducibly expressing p53, which undergo senescence after the removal of tetracycline from culture medium. Here, molecularly imprinted polymer nanoparticles were formed in the presence of induced (senescent) or uninduced (proliferating) cells such that exposed fragments of cell proteins in complex with polymeric networks were protected from proteolysis. Subsequent liquid chromatography tandem mass spectrometry (LC/MS-MS) analysis identified three putative senescence-specific proteins, which have not been analyzed in different cellular systems [85].

### 5.4. Soluble Markers

A proteomic approach was used to discover soluble biomarkers of senescence. Here, soluble proteins and exosomes were isolated from primary human lung fibroblasts induced to undergo senescence by different inducers. Samples were subjected to mass spectrometric analysis, followed by protein identification and quantification. The authors derived a proteomic database of senescence-associated soluble proteins and exosomal cargo defined SASP “Atlas” [76].

### 5.5. Other Markers

Senescent cells are characterized not only by quantitative proteomic changes but also by qualitative alterations such as covalent post-translational modifications. For instance, Itakura and colleagues, using lectin microarrays for glycan analysis, observed changes in both the cell surface and the intracellular glycan profiles in cellular senescence and human aging [86]. Baldensperger and colleagues analyzed post-translational modifications in different subcellular compartments during the aging process in a mouse cohort. The authors found that methionine sulfoxide, acetylation, formylation, and citrullination were the most abundant modifications in senescent cells and highlighted some interesting compartment-specific differences in protein modifications [87]. Another common age-dependent post-translational modification of proteins is oxidation, and a link between oxidative protein modifications and altered cellular metabolism in senescent human cells has been proposed [88]. Finally, advanced glycation end products (AGEs) have been proposed as potential aging biomarkers. AGEs represent non-degradable products of glycation of proteins, lipids, and nucleic acids, which are endogenously produced and accumulated during the normal aging process [89].

### 5.6. Markers Panels

In the face of the described efforts to establish a biomarker of cellular senescence that could be widely applicable, the most widely used approach for the identification of senescent cells still remains the analysis of panels of markers [21]. For instance, irreversible cell cycle arrest, decreased BrdU incorporation, upregulation of Cdkn2a, morphological changes, and SA-β-gal activity were detected in mouse embryonic fibroblasts [26]; morphologic alterations, gamma-H2AX and 53BP1 foci, and the SASP were analyzed in chemotherapy-treated cancer cell lines. Interestingly, these authors showed a big diversity in the quantitative values of different markers expressed in various cells treated with different drugs [5]. Seven different protein markers of senescence (i.e., pRb hypo-phosphorylation, lamin B1 loss, p53 phosphorylation status, accumulation of p53, p21, p16, and gamma-H2AX proteins) were detected by Jochems and colleagues in a panel of cell lines from four cancer types induced to undergo senescence by treatment with either etoposide or alisertib [18]. An increase in cells positive for TP53, CDKN1A/p21, gamma-H2AX, and galactosidase beta-1 was detected via IHC staining in a xenograft mouse model of glioblastoma [42]. Induction of senescence was confirmed by SA-beta-gal positivity and the mRNA expression of Cdk-inhibitors p16, p21, and p27 in both mouse and human prostate tumor cells in a screening for pro-senescence compounds. Furthermore, tumor volume and SA-beta-gal positivity were analyzed in in vivo experiments [28]. The analysis of such panels of markers is an onerous approach, which, although widely applied in cancer research, would not be easily translated in routine clinical settings. Hence, a shift to less-difficult and less-time-consuming techniques is required.

### 5.7. Morphology-Based Classifiers

Research has tried to overcome these technical challenges, and one recently pursued approach has been the development of image-based classifiers. The development of convolutional neural networks for medical research has greatly increased the accuracy of image classification and has enhanced its applications [90]. Since senescent cells are characterized by specific morphologic alteration, morphology-based classifiers have been developed. For instance, in order to identify a senescent state of endothelial cells, Kusumoto and colleagues developed a morphology-based convolutional neural network system and derived an automated quantitative scoring system for the classification of cellular senescence, referred to as Deep-SeSMo (deep-learning-based senescence scoring system by morphology). The initial input dataset used to train neural networks was represented by single-cell phase-contrast images of either hydrogen-peroxide-induced, camptothecin-induced, or replicative senescent HUVEC cells. Interestingly, the convolutional neural network trained on senescent HUVEC cells was also able to identify senescent human diploid fibroblasts, suggesting that morphologic characteristics of cellular senescence are shared by most types of senescent cells and can be used as specific markers [91]. Heckenbach and colleagues investigated the possibility of applying new computer vision techniques and machine learning methods to the analysis of senescence-specific alterations in nuclear morphology. Hence, using neural networks and nuclear morphology (nuclear area, aspect ratio, and convexity), Heckenbach and colleagues predicted a senescent status in replicative and ionizing-radiation-induced senescent human fibroblasts with up to 95% accuracy. When different time points were analyzed after ionizing radiation, the predictor was found to track the progressive development of the senescent phenotype. Trained on fibroblasts, the predictor was successfully applied to different cells, such as murine primary astrocytes and neurons, and more importantly, to hematoxylin-and-eosin-stained liver tissue from mice and human dermal biopsies. The authors infer that the shape of the nucleus provides a signal to indicate a senescent state in different cell types [13]. Similarly, nuclear morphology features of senescent cells were used to create machine-learning classifiers able to predict a senescent state in different cell types and tissues, in response to different senescent-inducing stimuli. These senescence classifiers and the derived tissue senescence score (TSS) were used to screen pro-senescence drugs for cancer therapy and to evaluate the efficacy of senolytic agents. Moreover, analyses of liver sections resected from patients with mild non-alcoholic fatty liver disease showed that the TSS also predicted senescence in human samples. Importantly, here, the classifiers were based on a small number of nuclear parameters, and the designed algorithms required less computational power than image-based neural networks, which simplifies downstream analyses and could make the classifiers clinically useable [92]. Deep learning technologies have also been applied to the detection of senescent mesenchymal stem cells through the development of a neural network system, which allowed for the automated detection, quantification, and analysis of senescent-related morphological changes [93]. Hence, these studies highlight that the application of artificial intelligence techniques could be an extremely useful tool in the study of cellular senescence, but a translation of artificial intelligence in routine clinic practice requires a wide diffusion across research and health professionals, which may not be familiar with deep learning. As recently pointed out, reviewing and explaining how artificial neural networks and deep generative models work could encourage its use in medical imaging [94].

### 5.8. Aptamers

Another technology recently applied for the identification of senescence biomarkers and for senotherapeutic drugs development is the aptamer. Aptamers are short oligonucleotide or peptide sequences that acquire a specific and unique three-dimensional structure depending on their primary sequence. Thanks to this specific three-dimensional conformation, aptamers can bind with high affinity and specificity to a range of cellular molecules and represent a powerful tool for the discovery of biomarkers, as recently reviewed by Li [95]. In addition, aptamers have been shown to distinguish post-translational modifications of target proteins, a property that makes them very attractive in the field of cellular senescence [96]. Using aptamer libraries, it is possible to select and enrich sequences that recognize distinct cellular targets through an iterative process known as SELEX [97]. In the context of senescence, Chen and colleagues applied a cell-based SELEX to identify single-stranded DNA aptamers specific for senescent synoviocytes, which represent an abundant fibroblast-like cell population in the synovium of the joints in osteoarthritis and play a causal role in the pathogenesis of the disease. This selection process led to the identification of three aptamer candidates (namely, CX1, CX2, and CX3) possessing good binding ability, and the CX3 aptamers was chosen for further investigation because of better serum stability. CX3 was used to functionalize liposomes, encapsulating two senolytic drugs (dasatinib and quercetin) for the specific targeting of senescent synoviocytes. Indeed, the CX3-functionalized liposomes showed high affinity for senescent synoviocytes, and intra-articular injection of CX3-functionalized liposomes effectively attenuates cartilage degradation in a mouse model of osteoarthritis [98]. DNA aptamers against AGEs, or against their receptor RAGE, have been synthesized and have been shown to inhibit AGEs cytotoxicity and to prevent the development and progression of AGEs-related diseases in different experimental models [99,100]. Aptamers have also been used for the development of complex molecular imaging and senotherapetic tools. For instance, in order to obtain real-time imaging of cellular senescence during cancer therapy, an aptamer-conjugated ratiometric fluorescent probe was designed and evaluated. In particular, an aptamer directed against the membrane protein L1CAM was used to recognize TIS cancer cells, and a beta-galactose linker, which could be cleaved by SA-beta-gal, was synthesized, which conjugated two cyanine fluorophores with a fluorescence resonance energy transfer (FRET) effect. Hence, this probe allowed for double recognition of TIS cells, first via aptamer-mediated cell recognition and then via senescent-specific activation, thereby increasing selectivity and specificity. The effectiveness of this probe was evaluated in cancer cells and tumor spheroids [101]. A similar approach was used to synthesize a senolytic agent for targeting senescent endothelial cells. Here, three functional groups that allow for three levels of recognition were associated: first, an aptamer targeting L1CAM was used to selectively recognize senescent endothelial cells; second, a beta-galactose linker enabled hydrolysis and activation via SA-beta-gal; third, EF24, which has been shown to inhibit anti-apoptotic pathways in senescent cells, was used as a senolytic agent. The authors validated this approach and showed that administration of the targeted senolytic efficiently eliminated senescent endothelial cells and foamy macrophages, contributing to the reduced plaque area in a model of atherosclerosis [102].

## 6. Conclusions

In recent decades, research has increased our knowledge about the role of therapy-induced senescence in cancer therapy. Initially, observations of chemo- and radiotherapy-induced senescence have suggested a positive effect of TIS due to permanent cell cycle arrest induced in cancer cells. Subsequently, the discovery of TIS escape and the characterization of tumor-promoting SASP shed negative light on TIS, which gained increasing attention. The ability of targeted therapeutic agents to induce senescence further expanded this research field. More recently, the development of transgenic approaches to eliminate senescent cells and the discovery of senolytic drugs have substantiated a detrimental function of senescent cancer cells. However, the application of senotherapy in cancer is hampered by the lack of reliable, cost-effective, and clinically useful methods by which to detect the induction of TIS in patients upon therapy. Recently, innovative imaging technologies and artificial intelligence systems have been adopted for the identification of TIS biomarkers, which have given promising results.

In conclusion, the identification and validation of TIS biomarkers in cancer is a stimulating research field and a priority, allowing for the routine clinical detection of TIS following anti-cancer therapy.

## Figures and Tables

**Table 1:** Senescence-inducing agents and experimental models for TIS used in reported studies.

Chemotherapeutic Agents	Targeted Drugs	In Vitro Model	In Vivo Model	References
	KAT6A/B inhibitors (WM-8014; WM-1119)	Mouse embryonic fibroblasts (MEFs); B cell lymphoma cells EMRK1184	EMRK1184 xenografts	[26]
	CDC7 inhibitor (XL413)	Human liver cancer cells (Hep3B, Huh7, HepG2, SNU182, SNU398, SNU449, Huh6, SK-Hep1); human non-transformed cells (BJ, RPE-1)	Huh7 and MHCC97H xenografts	[27]
	Retinoic-acid-receptor (RAR) agonist adapalene	Mouse embryonic fibroblasts (MEFs); mouse prostate tumor cells (TrampC1); human prostate tumor cells (PC3, LNCaP, 22RV1, LAPC4)	TrampC1 allografts; PC3 xenografts	[28]
Doxorubicin; bleomycin	CDK4/6 inhibitor palbociclib; p53 activator nutlin-3A	Human IMR-90 fibroblasts; mouse embryonic fibroblasts (MEF); human melanoma cells SKMEL-103; mouse melanoma cells B16-F10	B16-F10 allografts	[38]
Cisplatin	p53 activator nutlin; MEK inhibitor trametinib; CDK4/6 inhibitor palbociclib	Human liver cancer cells (HepG2, SK-Hep1); human lung cancer cells (A549, H460, H2030)	p53-restorable murine liver cancer cells orthotopic transplant model	[39]
Melphalan			Murine model of multiple myeloma (MM)	[40]
Doxorubicin		Human lung cancer cells (A549); Human breast cancer cells (MCF7)		[41]
Temozolomide		Human glioblastoma cells (A172, U87MG)	U87MG xenografts	[42]
	Aurora kinase inhibitors MLN8054 and MLN8237 (Alisertib)	Human melanoma cells (Hs294T, SK-Mel-5, SK-Mel-2 and SK-Mel-28); mouse melanoma cells MelA	Orthotopic implants of patients-derived melanoma	[43]
	MEK inhibitor trametinib; CDK4/6 inhibitor palbociclib	Fresh frozen sections of pancreas tumor tissue	Murine models of pancreatic ductal adenocarcinoma	[44]
	CDK4/6 inhibitor abemaciclib; CDK4/6 inhibitor palbociclib		MMTV-rtTA/tetO-HER2 transgenic mouse model of mammary carcinoma; patient-derived breast cancer xenografts	[45]
	CDK4/6 inhibitor abemaciclib	Human melanoma cells (GR39, UACC62, A2058); mouse cells B16F10	B16-F10 allografts	[46]
Cisplatin; irinotecan		Mouse ovarian cancer cells (UPK10, ID8)	Orthotopic implants of UPK10 cells	[47]
UV irradiation; mitomycin C	CDK4/6 inhibitor palbociclib.	Mouse embryonic fibroblasts (MEF); mouse melanoma cells (B16-F1, B16-F10)		[49]
Doxorubicin	p53 activator nutlin	4226 cells (from a spontaneous p53 WT MMTV-Wnt1 tumor); human breast cancer cells MCF-7	MMTV-Wnt1 mice	[52]
Mitoxantrone; X-ray; etoposide		Human fibroblasts (WI-38, IMR-90, HCA2); human prostate cells (PC-3, BPH1, RWPE1, DU145); human breast cells (MCF10A)	Tumor samples from patients with prostate cancer before and after mitoxantrone treatment	[53]
Etoposide; doxorubicin		Human lung cancer cells (A549); human breast cancer cells (MCF7); human pancreatic cancer cells (Panc-1)		[54]
Doxorubicin; paclitaxel; temozolomide; cisplatin		Mouse embryonic fibroblasts (MEF); mouse dermal fibroblasts (MDF); human dermal fibroblasts (HCA2, BJ)	p16-3MR mouse model	[57]
